# Peptoids successfully inhibit the growth of gram negative *E. coli* causing substantial membrane damage

**DOI:** 10.1038/srep42332

**Published:** 2017-02-14

**Authors:** Biljana Mojsoska, Gustavo Carretero, Sylvester Larsen, Ramona Valentina Mateiu, Håvard Jenssen

**Affiliations:** 1 Department of Science and Environment, Roskilde University, Universitetsvej 1, Postboks 260,4000 Roskilde, Denmark; 2 Department of Biochemistry, Institute of Chemistry. University of São Paulo, São Paulo, Brazil; 3 Department of Clinical Immunology, Naestved Hospital, Naestved, Denmark; 4 DTU CEN, Center for Electron Nanoscopy, Technical University of Denmark, Kgs Lyngby, Denmark

## Abstract

Peptoids are an alternative approach to antimicrobial peptides that offer higher stability towards enzymatic degradation. It is essential when developing new types of peptoids, that mimic the function of antimicrobial peptides, to understand their mechanism of action. Few studies on the specific mechanism of action of antimicrobial peptoids have been described in the literature, despite the plethora of studies on the mode of action of antimicrobial peptides. Here, we investigate the mechanism of action of two short cationic peptoids, rich in lysine and tryptophan side chain functionalities. We demonstrate that both peptoids are able to cause loss of viability in *E. coli* susceptible cells at their MIC (16–32 μg/ml) concentrations. Dye leakage assays demonstrate slow and low membrane permeabilization for peptoid 1, that is still higher for lipid compositions mimicking bacterial membranes than lipid compositions containing Cholesterol. At concentrations of 4 × MIC (64–128 μg/ml), pore formation, leakage of cytoplasmic content and filamentation were the most commonly observed morphological changes seen by SEM in *E. coli* treated with both peptoids. Flow cytometry data supports the increase of cell size as observed in the quantification analysis from the SEM images and suggests overall decrease of DNA per cell mass over time.

Traditional antibiotics gradually lose their efficacy against many pathogens due to fast resistance development by the microorganisms^1^. Moreover, the number of new antibiotics developed and approved is steadily decreasing^2^. Therefore there is an urgent demand for development of novel antimicrobial drugs that offer different treatment strategies. Antimicrobial peptides (AMPs), are a class of antimicrobial agents that have long served all living organisms in combating infectious diseases by their ability to kill or inhibit the growth of pathogens^3^,^4^. There is increasing evidence indicating that the primary direct antimicrobial activity of cationic antimicrobial peptides is through interaction with anionic bacterial surfaces^4^,^5^. On the other hand, the non-membrane disruptive mechanisms are often ascribed to the ability of certain AMPs to bind to intracellular targets and act on DNA, RNA and protein synthesis^3^,^4^,^5^. Other AMPs use a combination of membrane and intracellular activity, thus hampering their development as novel drug candidates with a specific target. Despite their effectiveness, one of the limitations of AMPs is their low bioavailability as a result of enzymatic degradation^6^. Therefore, AMPs have been subjected to various modifications to design novel classes of potent antimicrobials with improved stability and activity profiles, namely peptidomimetics. Peptoids, are oligomers of *N*-substituted glycines, a class of compounds that mimic the structure of peptides^7^. For more than 15 years, the research in peptoid synthesis and application, has dramatically increased due to their potential as compounds with broad biological activity profiles. Recently, studies have also demonstrated their antimicrobial properties^8^,^9^,^10^. Peptoids are less prone to enzymatic and proteolytic degradation and are more membrane permeable than peptides^6^,^11^. We and others, have previously demonstrated that the antimicrobial activities are retained upon translation of peptides into peptoids, thus suggesting that this class of antimicrobials acts via similar mechanisms^8^,^12^. Short cationic peptoids are shown to be able to decrease the number of viable bacterial cells, form pores, and bind to intracellular targets such as DNA^13^,^14^. Other have reported that the mechanism behind their bacterial killing is primarily associated with membrane disruption usually by pore forming mechanisms^13^,^15^. In an attempt to better understand the mechanism by which the most selective short cationic Peptoids 1 and 2, impair the ability of Gram-negative *E. coli* to divide, this paper describes their killing kinetics, membrane activity and their effect on *E. coli* morphology. Data clearly demonstrate that at peptoid MIC concentrations (Peptoid 1 MIC = 16 μg/ml, Peptoid 2 MIC = 32 μg/ml), the bacterial growth is successfully inhibited without any degree of membrane disruption, and a small degree of disruption is only detected at 4 × MIC.

## Results and Discussion

### *In Vitro* Antibacterial Activity

**Figure 1:**
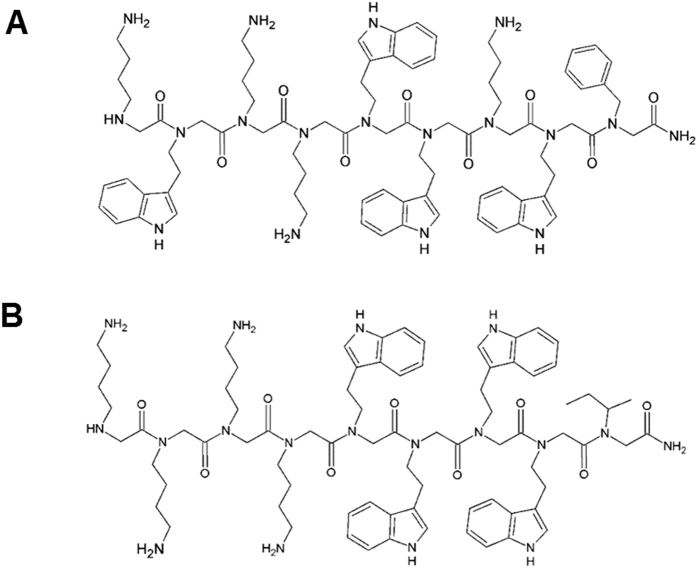
Chemical structures of peptoids. (**A**) Peptoid 1 and (**B**) Peptoid 2.

We have previously reported the broad spectrum activity of a library of cationic tryptophan rich peptoids against six bacterial strains and compared those activities with the ones of similar antimicrobial peptides^12^,^16^. In addition, the hemolytic activity and cytotoxicity against HeLa cells were analysed, altogether demonstrating good selectivity profiles for these peptoids. Two peptoids showed good selectivity index values with respect to their activity against *E. coli* and their haemolytic values and were therefore chosen for further studies (Table 1). Both peptoids have relatively short sequences (9 residues, Fig. 1) and exhibit MIC ( ≤ 22.2 μM) values that are comparable to that of the antimicrobial peptide indolicidin (Table 1) and are within the range of other antimicrobial peptoids reported in the literature^8^,^17^.

### *E. coli* killing kinetics

**Figure 2:**
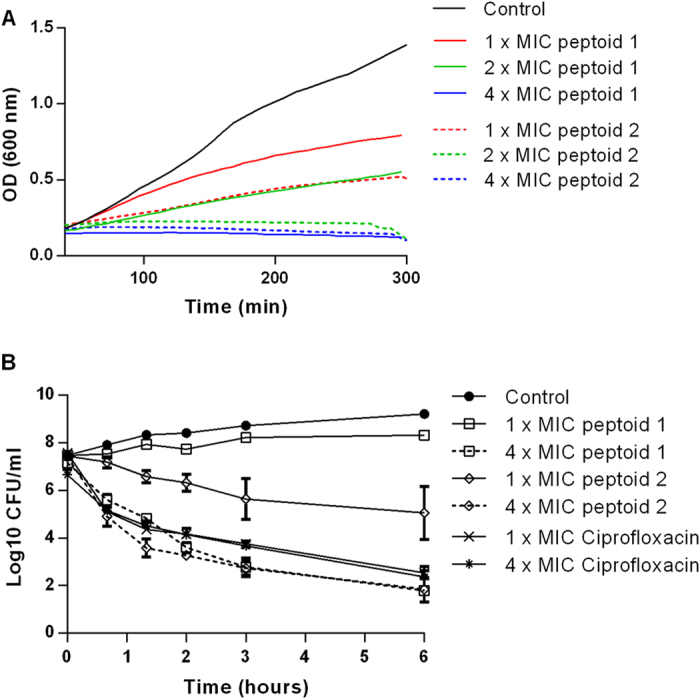
Killing kinetics. (**A**) Optical density measurements at 600 nm of exponentially growing *E. coli*, with and without Peptoid 1 at 1 × MIC (16 μg/ml), 2 × MIC (32 μg/ml) and 4 × MIC (64 μg/ml) and Peptoid 2 at 1 × MIC (32 μg/ml), 2 × MIC (64 μg/ml) and 4 × MIC (128 μg/ml) concentrations. (**B**) Time-kill study of *E. coli* ATCC 25922 challenged with 1 × MIC (16 μg/ml, 32 μg/ml and 0.05 μg/ml) and 4 × MIC (64 μg/ml, 128 μg/ml and 0.2 μg/ml) concentrations of Peptoids 1 and 2 and ciprofloxacin, respectively. For both experiments *E. coli* cultures are in log phase at 2–4 × 10^7^ CFU/ml. Data represents mean and SEM of 3 independent experiments.

In order to differentiate between bacteriostatic and bactericidal mode of action of peptoids 1 and 2, *E. coli* cultures were challenged with different concentrations in a period of 6 hours and growth inhibition patterns were compared. Data supports the inhibition capacity of both peptoids which show clear concentration dependent inhibition of *E. coli*. Both Peptoids 1 and 2 at 1 × MIC concentrations exhibit bacteriostatic mode of action on *E. coli* growth (Fig. 2A,B). The slow killing for Peptoids 1 and 2 may indicate that at MIC concentration these peptoids target intracellular metabolic processes that cause bacterial growth inhibition. With respect to this observation, delayed killing kinetics have been observed for AMPs that target intracellular compartments^18^,^19^. These could be inhibition of DNA synthesis or cell filamentation as observed for bacteriostatic antimicrobial compounds^20^. At 2 × MIC concentrations different killing kinetics are observed, where Peptoid 2 acts via bactericidal mechanism (Fig. 2A). The observed difference may be partly due to the different hydrophobicity profiles of these peptoids measured by retention times on HPLC. Here, Peptoid 2 is globally more hydrophobic and this property could enhance its insertion into bacterial membranes. Similar killing kinetic profiles as Peptoid 2 have been previously observed for other antimicrobial peptidomimetics^13^,^21^,^22^,^23^. At 4 × MIC concentrations, both peptoids show significant bactericidal behaviour which is marginally higher than that shown for ciprofloxacin, an antibiotic that prevents DNA replication, recombination and repair (Fig. 2B)^24^. The fast killing kinetics is still not higher than that reported in the literature for polymyxin B, a fast permeabilizing antimicrobial peptide, where upon reaching a threshold concentration, the accumulated peptide molecules of polymyxin B can rapidly cause bacterial death^25^.

### Quantification of membrane permeabilization

**Figure 3:**
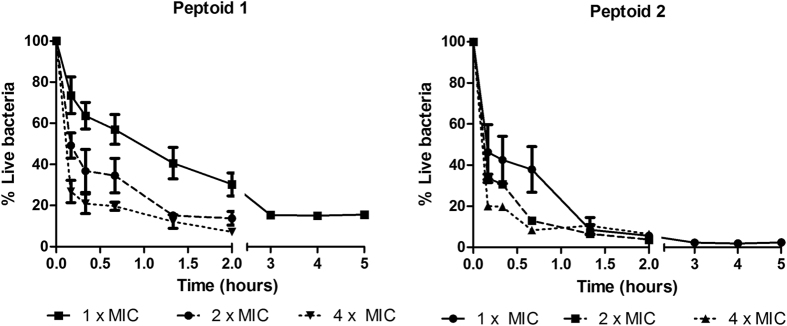
Quantification of viable bacteria using Live/Dead staining. Percentile live bacteria treated with Peptoid 1 at 1 × MIC (16 μg/ml), 2 × MIC (32 μg/ml) and 4 × MIC (64 μg/ml) and Peptoid 2 at 1 × MIC (32 μg/ml), 2 × MIC (64 μg/ml) and 4 × MIC (128 μg/ml) (Eq. 1 see Experimental Section). 100% is set as the value from untreated samples at time zero. Results are from 3 independent experiments with SEM error bars.

The mechanism of action of antimicrobial peptides and related mimetics that carry net positive charge and moderate hydrophobicity on bacteria is generally associated with fast killing kinetics as a result of membrane permeabilization^18^,^23^,^26^,^27^,^28^. To investigate whether the bactericidal effect observed from the killing kinetics experiments is related to the ability of the peptoids to disrupt bacterial membranes, we calculated the percentile of viable and non-viable cells using Live/Dead quantification assay. In this assay two nucleic acid dyes, syto 9 green and propidium iodide (PI) red, that have different membrane permeabilities were used as indicators of live bacteria with intact membranes versus dead bacteria with compromised membranes, respectively. Peptoid 1 caused gradual decrease of the number of PI stained cells at 1 × MIC concentration. In response to Peptoid 2 treatment, 90 percent of *E. coli* showed high membrane permeabilization within one hour of treatment, that supports the observation for the higher potency exhibited by this peptoid from the killing kinetics experiments (Fig. 3). Importantly, the activity of both peptoids remains stable after 5 hours of incubation. This is in contrast to the activity observed for the peptide from which these peptoids are derived, where re-established growth is observed within 2 hours of peptide administration^16^.

### Release of fluorescent dyes from different liposome compositions

**Figure 4:**
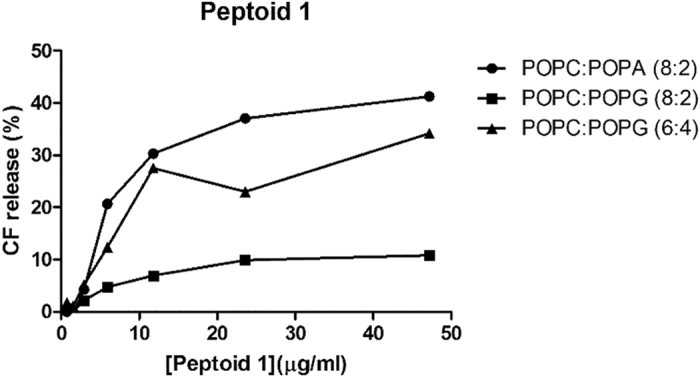
Carboxyfluorescein release for Peptoid 1. Carboxyfluorescein release over time from three liposome compositions at different Peptoid 1 concentrations is shown.

Two fluorescent dye leakage assays were used to assess the disruptive ability of peptoids. The two assays use similar fluorescent dyes, carboxyfluorescein (CF) and calcein to measure the leakage from the liposomes. These two assays offer information on the electrostatic interaction with membranes mimicking bacterial surfaces and interaction with membranes with different fluidities. Bacterial membranes have a highly negative transmembrane potential (approximately −120 mV) in contrast to the weak membrane potential maintained by mammalian cells^29^. Thus they attract positively charged compounds such as cationic peptoids. Therefore, to test the existence of a specific interaction between Peptoid 1 and the glycerol polar headgroup of palmitoyl-2-oleoyl-*sn-glycero*−3-phospho-(1′-*rac*-glycerol) (POPG) or, if the interactions are purely electrostatic, carboxyfluorescein (CF) leakage assays from liposomes of 1-palmitoyl-2-oleoyl-*sn*-glycero-3-phosphocholine (POPC) containing 20 mol % of 1-hexadecanoyl-2-(9Z-octadecenoyl)-*sn-glycero*-3-phosphate (POPA) or POPG were performed initially. Both POPA and POPG are negatively charged phospholipids but with different polar headgroup structures; while PA phospholipid only has a phosphate group linked to C3 of the glycerol moiety, PG has a phosphoglycerol group at this same position. Liposomes containing 20 mol % of negatively charged POPA showed to be more susceptible to Peptoid 1 when compared to liposomes with 20 mol % of POPG (Fig. 4). This indicates that there is no specificity in peptoid binding to PG. Again, the greater extent of dye release from liposomes with 40 mol % of POPG compared to 20 mol % of POPG shows the importance of electrostatic interactions between the peptoid and liposomes and that its activity increases with the increase of overall net surface charge (Fig. 4).

**Figure 5:**
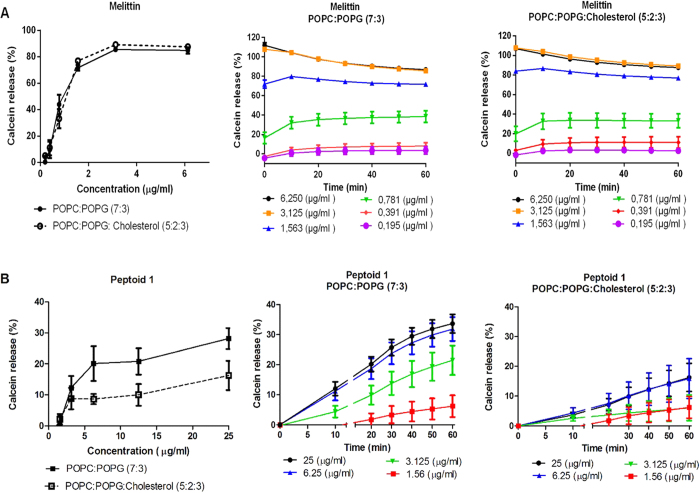
Calcein release from two liposome compositions, POPC:POPG (7:3) and POPC:POPG:Cholesterol (5:2:3) for (**A**) Melittin and (**B**) Peptoid 1. Calcein release as a result of different concentrations and concentration dependent release over time is shown. Results are from minimum of 3 independent experiments with SEM error bars.

Furthermore, besides electrostatic interactions, hydrophobic side chain functionalities contribute greatly to the peptoid–membrane interactions. Tryptophan-like residues have strong membrane disruptive activities due to their ability to interact with the membrane interface. This activity is assigned partly due to the flat and rigid structure of the indole ring that may favour alignment of the side-chain in the membrane interface region^4^. To evaluate the permeability of the peptoids to membranes, we used the calcein leakage assay where calcein was encapsulated in two different combinations of selected lipids POPC:POPG (7:3) and POPC, POPG and Cholesterol (5:2:3). The membranes of mammalian cells have sterol molecules such as Cholesterol in their membrane compositions and this property can prevent peptoid insertion in the lipid bilayers. We used membrane composition containing POPC, POPG and Cholesterol (5:2:3), as reported previously, to mimic membrane environment with decreased fluidity while retaining negative charge to ensure peptoid binding^16^,^30^. In addition, the liposomes composed of POPC, POPG and Cholesterol also mimic membrane compositions of some of the membranes of tumour cells and neurons, even though POPG is not found as a part of the composition of mammalian membranes^31^. The total amount of released calcein was monitored and the degree of membrane permeabilization of the peptoids was calculated using Equation 2. Distinct tendency to lyse liposomes mimicking bacterial membranes (POPC:POPG) over liposomes mimicking less fluidic membranes, was observed for Peptoid 1, in contrast to the indiscriminative membrane permeabilization activity of the control peptide melittin that acts via the toroidal pore formation mechanism (Fig. 5A,B)^32^. Membrane permeabilzation is expected for the peptoids in this study because of their structural compositions. Short tryptophan rich cationic peptides have been reported to exhibit high membrane activity on *E. coli* where the peptide with the highest tryptophan content (2 W) and charge (+9) showed highest percentage of membrane permeabilization (approximately 60%)^33^. Similarly, the peptoids in this study are rich in charged (+4) and tryptophan-like residues (4 W) which are expected to contribute significantly to the observed membrane permeabilizing properties. The permeability of peptoid 1 was greater for liposome compositions that mimic bacterial membranes (Fig. 5). Maximum calcein leakage (<30%) was observed from sub MIC concentrations of Peptoid 1 and an increase in the peptoid concentration from 6.25 to 25 μg/ml did not contribute to an increase in the calcein release from these liposomes (Fig. 5B), thus indicating that Peptoid 1 only has a weak membrane activity. The membrane activity is also much lower than the one observed for structurally similar GN-4 antimicrobial peptide we have published previously^16^. The unique physiochemical properties of peptoids such as loss of hydrogen donation and backbone chirality may be expected to impact the membrane interactive properties when compared with peptides. However, not all antimicrobial peptides insert and disrupt the membranes or form pores. Studies on mechanism of action of cationic tryptophan rich peptides have shown that they either primarily lyse the membrane of Gram-negative bacteria by pore formation or they translocate and act on internal targets^34^. The overall total thickness of the membrane of Gram-negative bacteria varies from 30–50 nm. In order for a peptide or peptidomimetic to form a pore in the membrane, it has to be long enough to span the membrane e.g. barrel-stave model. Some peptides are too short to span the membranes^35^,^36^. They usually induce pore formation via a toroidal model by stabilizing the lipid pore and forming a peptide-lipid complex^37^. Indolicidin, a short (13 residue), tryptophan rich, extended structure cationic peptide, is able to cause leakage of calcein and carboxyfluorescein from negatively charged liposomes via non-membrane pore formation mechanism, but rather via translocation of dye molecules across the membrane in the form of dye-peptide complexes^35^.

Concentration dependent calcein release was observed from liposomes containing Cholesterol, but with a significantly slower release rate and a lower threshold, which is in agreement with the low toxicity reported for Peptoid 1 at concentration as high as 4 × MIC. Calcein leakage in a concentration dependent manner is also observed for antimicrobial peptides^38^. This concentration dependent activity of antimicrobial peptides has been explained by a two-state model^37^. This model illustrates initial binding of peptide monomers parallel to the plane of the membrane. The peptides are then dispersed all across the membrane without causing pore formation. As the concentration of the peptide increases, thinning and formation of transmembrane pores takes place^37^. Similar membrane thinning has been reported for indolicidin^35^,^39^. Our findings suggest that the release of calcein upon addition of Peptoid 1 is concentration dependent and significantly lower than the total calcein release observed for melittin (Fig. 5A,B)^40^. The total dye leakage together with the results on the bacterial viability quantification at concentrations around 1× and 2 × MIC of Peptoid 1, are in agreement with the killing kinetics experiments indicating that the growth inhibition is partially exerted by some degree of membrane damage that does not include toroidal pore formation.

### Changes in Membrane Morphology of bacteria upon peptoid treatment

**Figure 6:**
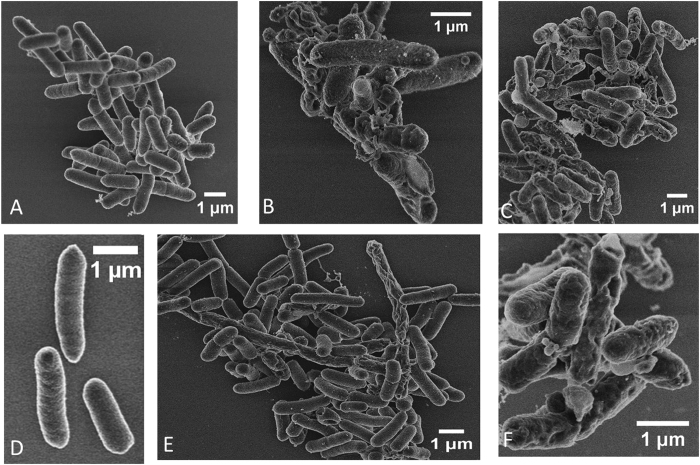
Scanning electron micrographs of *E. coli* ATCC 25922 untreated and exposed to Peptoid 1 (at concentrations corresponding to 1× and 4 × MIC for a period of 1 and 4 hours. Cultures in log phase at 2–4 × 10^7^ CFU/ml. (**A**) Untreated *E. coli* at 1 hour. (**B**) *E. coli* treated with 1x MIC (16 μg/ml) at 1 hour. (**C**) *E. coli* treated with 4x MIC (64 μg/ml) at 1 hour. (**D**) Untreated *E. coli* at 4 hours. (**E**) *E. coli* treated with 1 × MIC (16 μg/ml) at 4 hours, and (**F**) *E. coli* treated with 4 × MIC (64 μg/ml) at 4 hours.

**Figure 7:**
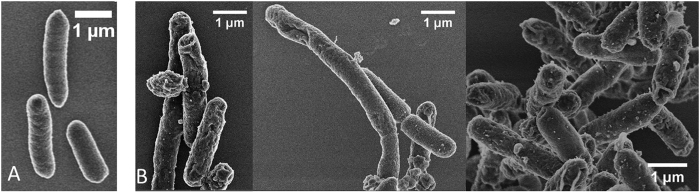
Scanning electron micrographs of *E. coli* ATCC 25922 untreated and exposed to Peptoid 2. Cultures in log phase at 2–4 × 10^7^ CFU/ml. (**A**) Untreated bacteria at 4 hours, (**B**) Bacteria treated with 4 × MIC at 4 hours.

**Figure 8:**
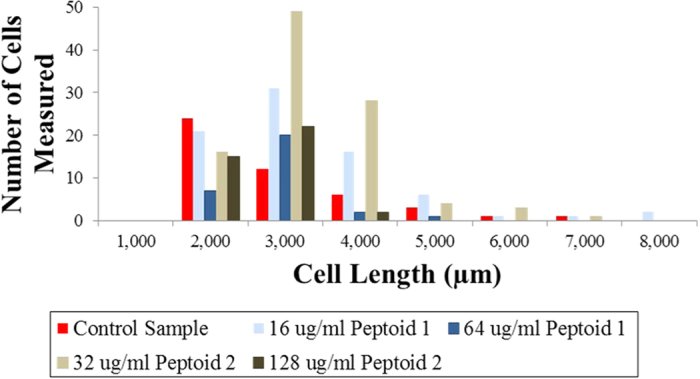
Quantification of *E. coli* size distribution. Data from 1 hour incubation with and without 1× (16, 32 μg/ml) and 4 × MIC (64, 128 μg/ml) concentrations of Peptoids 1 and 2, respectively.

*E. coli* were inspected using scanning electron microscopy to visualize any morphological changes (membrane disruption, cell elongation, etc.) after peptoid treatment. Untreated bacterial cells appear with smooth surfaces whereas considerable roughening and leakage of cytoplasmic content is observed for peptoid treated bacteria (Figs 6 and 7). The SEM micrographs show membrane damage upon treatment with both peptoids at 1× and 4 × MIC, with a more pronounced effect at 4 hours (Figs 6 and 7). These cellular changes are related to the growth inhibitory activities observed at 1× and 4 × MIC concentrations. Membrane disintegration is expected for these peptoids due to the presence of four tryptophan like side chains which are strongly associated with high affinity for the interfacial region of bilayers^41^. Similar changes in bacterial surfaces, such as membrane damage and blebs have been observed both for antimicrobial peptides and for metal oxide nanoparticles with negative surface potential as a result of their strong interaction with *P. aeruginosa* and *E. coli* membranes, respectively^5^,^42^. Bleb morphology in *E. coli* has also been reported for the human antimicrobial peptide defensin 5, where the cause of bleb formation was localization of this peptide at the site of cell division and site poles indicating that the antibacterial activity is exerted in the cytoplasm^43^. Additionally, *E. coli* treated with the cationic antimicrobial peptide, gramicidin, illustrated a high degree of blister and dimple formation^44^. Filamentation, a continuous cell elongation which does not result in cell division, is often observed in bacteria as a result of various stress responses such as those to beta lactams and fluoroquinolones antibiotics^45^,^46^. Bacterial filamentation has been reported as a consequence of induction of SOS response mechanism which is responsible for regulation of DNA damage repair^47^,^48^. Elongated bacteria were observed among the affected bacterial population indicative of inhibition of bacterial cell division (Figs 6E and 7B). In addition, from the quantitative data, the majority of the *E. coli* with cell length of 3 μm were those treated with 1 × MIC concentration of peptoid 2 (Fig. 8). Antimicrobial compounds that act via bacteriostatic mechanisms (e.g. inhibition of protein and RNA synthesis) have been shown not to induce filamentation or increase cell size^49^. Therefore the increase in cell size in bacteria treated with peptoids could be due to possible inhibition of DNA processes inside the cell. These results indicate that peptoids can destabilize the membrane of *E. coli* causing pronounced damage that does not always lead to complete lysis and cell death, but to cell elongation.

### Changes in size and DNA content

**Figure 9:**
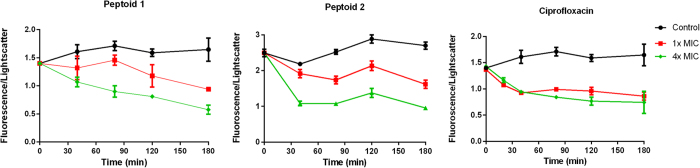
Flow cytometry data of exponentially growing *E. coli* exposed to Peptoids 1 and 2 and ciprofloxacin at concentrations corresponding to 1× and 4 × MIC. Fluorescence corresponds to cellular DNA content and lightscatter is equivalent to the bacterial cell size, thus fluorescence/lightscatter is a measure of the DNA content per cell mass. Results are from 3 independent experiments with SEM error bars.

We used flow cytometry to measure the changes in cell size and nucleic acid content reflected by increased light scatter and fluorescence, respectively. Exponentially growing cells were challenged with both peptoids and ciprofloxacin and stained with propidium iodide (PI) cell impermeant dye. PI stains nucleic acids in cells with damaged membranes. Untreated cells retain constant DNA per cell size (Fig. 9). Bacteria treated with ciprofloxacin appear significantly bigger with overall decrease of DNA per cell size. Certain quinolone antibiotics such as ciprofloxacin or norfloxacin interact with DNA gyrase and topoisomerase IV causing DNA replication fork arrest and induction of SOS response in *E. coli*. This leads to inhibition of cell division and filamentation^50^,^51^,^52^. Ciprofloxacin is also shown not to affect the membrane integrity of *E. coli* even at high concentrations^46^,^53^. The bactericidal effect of ciprofloxacin demonstrated in the killing kinetics experiments is in agreement with the decrease of DNA per cell mass obtained from the flow cytometry measurements. This effect is thought to be related to the release of free DNA ends from DNA gyrase-ciprofloxacin complex that ultimately leads to chromosomal DNA fragmentation^20^,^54^. The fluorescence signal from PI binding to nucleic acids in the cells may therefore indicate a different pathway of membrane disturbance to be involved in making the bacteria permeable to the dye^55^. Investigation into the effect of the peptoids on the cell size and DNA content demonstrated that cell size increased considerably with an increase in concentration for both peptoids. The overall effect of the peptoids was a substantial decrease in DNA per cell mass, which was concentration dependent and it occurred at faster rate for peptoid 2 when compared to that of ciprofloxacin. A similar dose dependent interaction between peptoid and bacterial plasmid DNA was confirmed using a gel retardation assay (Fig. 2, see Supplementary Material). At 1 × MIC the inhibition pattern was somewhat less pronounced for the peptoids in regards to ciprofloxacin demonstrating a possible distinctive antibacterial effect.

## Conclusions

Many antimicrobial peptides have been referred to as “dirty drugs” due to their ability to act via multiple mechanisms which involve membrane permeabilization and inhibition of intracellular processes^36^,^56^. While dissecting the mode of action of lysine and tryptophan rich peptoids that mimic the structure of antimicrobial peptides, we have demonstrated that the growth inhibition ability of peptoids that show different killing kinetics is most likely based on the different charge density distribution and overall hydrophobicities. We show that the killing mechanism for peptoid 1 is not fully attributed to its ability to cause leakage from the membranes as observed in the dye leakage assays. However, the killing mechanism is definitely supported by some degree of membrane damage exerted by this peptoid. Higher degree of bacterial lysis through membrane damage is observed for peptoid 2. The peptoids exhibited potent killing kinetics which are not explained solely by their membrane permeabilization ability, measured by calcein leakage from lipid vesicles mimicking bacterial membranes. However, the high degree of membrane disturbance measured by Live/Dead assay is not directly translated into a decrease of viable cells. The membrane disruptive ability is also supported by scanning electron micrographs of *E. coli* treated with both peptoids. Although the mechanism behind the observed increase in size is not clear, it can be reasonably assumed that besides the peptoids effect on the outer membrane they enter the cytoplasm and bind to internal targets thus inhibiting DNA, RNA or protein synthesis. Taken together we propose that these peptoids act via combined mechanism of action involving membrane disruption with probable intracellular targets. Further investigations are needed to support their effects via intracellular targets. The peptoids in this study have low toxicity, high stability and low cost of synthesis, properties which contribute to their high potential as new peptidomimetics with therapeutic applications.

## Experimental Section

### Materials and Bacterial strains

The strains used in the present study were from our laboratory strain collection. *Escherichia coli* (ATCC 25922) was obtained from the American Type Culture Collection (ATCC, Rockville, Md.). Other strains include *Pseudomonas aeruginosa* PAO1 H103 strain and Liverpool epidemic clinical strain H1027, and *Escherichia coli* clinical isolate expressing extended spectrum β–lactamases (ESBL)^57^. All tested bacterial strains are categorized as biohazard level 2 pathogens.

### Peptoid synthesis

Peptoids were synthesized using standard submonomer solid-phase synthesis, as described previously^12^.

### *In vitro* susceptibility studies

Minimum inhibitory concentrations (MIC) for the peptoids have been measured as described previously^12^.

### Killing kinetics

The kinetics of antimicrobial activity against *E. coli* (ATCC 25922) were assessed at a peptoid concentration corresponding to 1×, 2× and 4 × MIC. Briefly, an overnight culture of bacteria was diluted 1:50 in fresh Mueller Hinton broth and regrown to an OD_600_ of 0.4 before diluting to a turbidity of 0.1. The bacterial suspension was added to a 96-well polystyrene flat bottomed plate containing the peptoid of interest in addition to known antibiotics. The plate was incubated without shaking at 37 °C for 180 minutes. Samples (100 μl) were taken at time 20, 40, 80, 120 and 180 minutes and diluted in ice cold 0.9% NaCl from which 100 μl was plated on LB agar plates. The plates were incubated for 18–24 hours at 30 °C and colony forming units (CFU) were counted. Sterility control was performed by plating 100 μl of MH broth and 0.9% NaCl. Plates where no detectable bacterial growth was observed were left for additional 18 hrs incubation.

### Live/Dead Staining

Overnight culture of *E. coli* ATCC 25922 was diluted 100-fold in fresh MH broth and allowed to grow until it reached OD_600_ of 0.1. Duplicate samples of 1 ml each were transferred to eppendorf tubes and pelleted at 10.000 g for 8 minutes. One sample was resuspended in 1 ml 0.9% NaCl and the other in 50 μl 0.9% NaCl followed by addition of 950 μl 70% isopropyl alcohol to kill the bacterial. The tubes were left for 1 hour on ice with manual shaking every 15 minutes. The tube containing dead bacteria was pelleted at 12.000 rpm for 8 min and resuspended with 1 ml 0.9% NaCl to remove the isopropyl alcohol. Five different ratios corresponding to 0, 10, 50, 90 and 100% of live bacteria were prepared to obtain the standard curve for the analysis of bacterial viability when challenged by antimicrobials (Fig. 1, see Supplementary Material). Briefly, 100 μl of the staining solution (3 μl of 3.34 mM SYTO 9 and 3 μl of 20 mM propidium iodide in water) was added to 100 μl of the different bacteria ratios containing wells and incubated in dark for 15 minutes before reading on multi-detection macroplate reader Synergy HT. The green fluorescence (SYTO 9) corresponds to the amount of live bacteria and was excited at 485 nm and the emission detected at 528 nm. The red fluorescence (propidium iodide) was excited at 530 nm and detected at 645 nm which correspond to the amount of dead bacteria in the sample. Bacteria suspension challenged with antimicrobial for viability analysis were prepared by incubating 90 μl of bacteria (OD_600_ of 0.1) and 10 μl antimicrobials for 5 hours during which period samples were extracted in PCR tubes at 0, 10, 20, 40, 80 and 120 minutes and immediately put on ice. All PCR tubes were pelleted at 10.000 g for 8 minutes before being resuspended in cold 0.9% NaCl and placed on ice again. The content of each PCR tube was then added to individual wells of flat bottomed polystyrene 96 microtitter plate and mixed with 100 μl staining solution. After 15 minutes incubation in dark the fluorescence was measured using multi-detection microplate reader Synegry HT. The percentage of living cells was calculated using Equation 1 below.


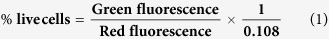


### Liposome preparation

Two different compositions of large unilamellar vesicles (LUVs) were made. To mimic the composition of bacterial membranes 1-palmitoyl-2-oleoyl-sn-glycerol-3-phosphoglycerol (POPG) and 1-palmitoyl-2-oleoyl-sn-glycerol-3-phosphocholine (POPC) in a molar ratio of 7:3 was prepared. The second composition, a simple mimic of mammalian membranes, was made from POPC:POPG:Cholesterol in a molar ratio of 5:2:3. The indicated lipids were purchased from Avanti Lipids (Alabaster, Alabama). The lipid mixtures were initially mixed in chloroform, after which the solvent was removed under low pressure at 40 °C in a rotary evaporator over 2 hours. Ethanol (99.9%) was frequently added to remove residual organic solvent. The lipid mixture was then dissolved in 4 ml HEPES buffer (10 mM HEPES, 150 mM KCl, 0.03 mM CaCl_2_, 0.01 mM EDTA. pH 7.4) with 20 mM calcein (C0875, Sigma), for calcein containing liposomes. Multiple mixing and sonication steps for 5 minutes were performed to avoid aggregation. Subsequently, the lipid mixture was vigorously whirl-mixed every 10 minutes over a course of 1 hour and finally left at room temperature for an additional hour to allow the lipids to anneal. LUVs were prepared by extruding the lipid mixtures through double stacked 100 nm filters, a total of 10 times using Nitrogen powered extruder and 30 bars pressure. Calcein containing liposomes were loaded on Sephadex G-50 columns to separate encapsulated from free calcein and the elution was done using calcein free HEPES buffer. For verification of the size of the liposomes, dynamic light scattering (DLS) measurements were obtained on a Zetasizer Nano ZS (Malvern, Worcestershire, UK). Monodisperse liposomes of a size of 110 nm was measured by Malvern DTS v. 5.10 software.

### Liposome quantification

Quantification of lipid content was performed using standard protocols described previously^58^. Initially, standard curve was obtained using 0, 20, 40, 60, 80, and 100 nmols of standard potassium phosphate solution (1 mM). Briefly, the tubes containing 0, 20, 40, 60, 80 and 100 μl of potassium phosphate buffer were dried in a heating block at 120 °C and 400 μl of perchloric acid (HClO_4_) was added. For determination of the lipid content of the prepared liposomes, 50 nmols were dried and 400 μl of perchloric acid was added. Digestion of the lipids was achieved by heating the suspension at 180 °C for up to 2 hours. After all samples have been cooled to room temperature, 1 ml of MilliQ water is added, followed by addition of 400 μl of ammonium molybdate (1.252 g/100 ml). All the tubes are vortexed and 400 μl of freshly prepared ascorbic acid (3% w/v) is added. The tubes are put to boiling water for 10 minutes and after they cool down, 1 ml is transferred to a 1 cm cuvettes and absorption is read using standard spectrophotometer at 797 nm. Using the standard curve equation, the concentration of liposome suspension was calculated before use.

### Calcein release assay

Calcein release was done in a 96-well plate with shielded wells (MicroWell 96 optical bottom plate, NUNC, Roskilde, DK) as previously described^16^. Briefly, peptoids were diluted in 10 mM HEPES buffer and 100 μl were added to wells followed by addition of 80 μl of 45 μM liposomes suspension for a final liposome concentration of 20 μM. Immediately after addition the fluorescence was read using multi-detection microplate reader Synergy HT at an excitation wavelength of 485 nm and an emission wavelength of 520 nm over a course of 1 hour at 37 °C. Maximum calcein release was acquired using 10% Triton X-100 and release following peptoid exposure was calculated using equation 2, where F and F_o_ represent the initial and the final levels of fluorescence before and after peptoid addition, respectively, and F_max_ is the fluorescence level after complete disruption of liposome by addition of detergent, 10% Triton X-100.


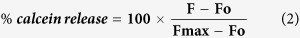


### Carboxyfluorescein leakage assay

The permeabilizing activity studies of the peptides upon model membranes were performed as previously described^59^. This test is based on the property of self-quenching of carboxyfluorescein when in high concentrations, and high quantum yield when diluted. Stock solutions of the lipids were prepared in chloroform:ethanol mixture. Lipid films were obtained by evaporating the solvent from pre aliquoted mixtures of the stock solutions under a stream of N_2_ and submitted to vacuum for 2 hours. The lipid film is then resuspended in a 50 mM solution of carboxyfluorescein with Tris-HCl 20 mM and NaCl 300 mM and pH 7.4. The lipid suspension is extruded through two stacked polycarbonate filters with 100 nm pore size (Nuclepore, Maidstone, UK) to obtain the large unilamellar vesicles (LUV). At this stage, the suspension has 50 mM of carboxyfluorescein in the internal compartment of the LUV at the external environment. The LUV are separated from non-encapsulated carboxyfluorescein by a process of gel-filtration chromatography on a pre-packed Sephadex G-25 mini-column (GE Healthcare, Buckinghamshire, UK) equilibrated with the buffer. Lipids were further quantified by the Rouser method^58^. The total lipid concentration in the samples was 20 μM. The carboxyfluorescein release was determined same as calcein release, described earlier (Eq. 2).

### Scanning electron Microscopy (SEM)

Overnight culture (approx. 18 h at 37 °C) of *E. coli* ATCC 25922 was diluted 1:50 in fresh MH broth and regrown to an OD_600_ of 0.4 before diluting it to a turbidity of 0.1. Bacteria were treated with 1× and 4 × MIC concentrations of peptoids GN-2 Npm_9_ and GN-2 Nlys_1–4_ Ntrp_5–8_ prepared in MH Broth for 4 h at 37 °C in a microtitter plate. Samples were taken at two time points, one at 1 h and the second at 4 h incubation. To ensure enough cells for analysis a volume of 600 μl (3 × 200 μl) was polled into 1.5 ml Eppendorf tubes. The sample was then chemically fixed in 3% glutaraldehyde in MHB, pH adjusted to 7.3 at 4 °C for 16 hrs. After washing 3 times in distilled water, for 10 min each time, the sample was stained with 1% OsO_4_ at 4 °C for 16 hrs. Next, the sample was washed again, 3 times, and dried in ethanol series at 25 °C for 10 minutes for each ethanol step (30, 50, 70, 80, 90 and 100% ethanol). The 100% ethanol was replaced with acetone in three 10 minute steps: 30, 50 acetone and 100% acetone. After the acetone step, the sample was further dried in a Leica EM CPD300 onto a square piece of silicon wafer. The silicon substrate with the dried sample was attached onto an aluminium stub with a double sided C tape, and the sample was coated with 2 nm Pt in a Cressington 208HR High Resolution Sputter Coater. The sample was then imaged in an FEI Helios dual beam scanning electron microscope by monitoring the secondary electron signal at 2 keV and 43 pA with the through the lens detector. The size analysis was performed using the NIH public domain Image J.

### Flow cytometry

Overnight culture of *Escherichia coli* ATCC 2592 was diluted 1:50 in fresh MH broth and allowed to grow until an OD_600_ of 0.1. This suspension is further 10-fold diluted and regrown till OD_600_ of 0.1 to ensure a uniform bacterial population. A volume of 90 μl of bacterial suspension was loaded to a flat bottomed 96-well Greiner plate. After extraction at zero minutes, 10 μl of the peptoid and antibiotics corresponding to 1× and 4 × MIC concentrations was loaded to the bacteria containing wells for a total volume of 100 μl. Samples were collected at 20, 40, 80,120 and 180 minutes and put on ice. The plate was sealed and incubated at 37 °C between sample extractions. Samples from wells containing bacteria without peptoids or antibiotics were taken at each time point. The samples were, whenever appropriate, centrifuged at 10.000 × g for 5 min at 4 °C. The supernatant was carefully removed and the bacteria were resuspended in 100 μl 10 mM Tris HCl pH 7.4. 1000 μl of ice cold 77% Ethanol was added before storing the samples at 4 °C until analysis. Rifampicin/Cephalexin samples were prepared by collecting 200 μl from the wells containing bacteria and the appropriate antimicrobial in an E-tube containing 45 μl of a mixture of Rifampicin (300 μg/ml) and Cephalexin (36 μg/ml). The tube was placed in a 37 °C shaking water bath for 2½ hours after which the samples were treated in the same way as the other samples before storing them at 4 °C. To monitor the bacterial growth the plate was read before each sample was extracted in a multi-detection plate reader Synergy HT. The following day, the samples were centrifuged at 10.000 × g for 5 min and the supernatant was carefully removed. The samples were stained for flow cytometry analysis with 140 μl of staining solution (90 μg/ml Mitramycin and 20 μg/ml Ethidium Bromide in 10 mM Tris pH 7.4, 10 mM MgCl_2_). For flow cytometry analysis A10 Bryte Flow Cytometer was used and around 20.000 events were included.

### DNA binding assay

el retardation experiments were performed by mixing 100 ng of bacterial plasmid DNA (pBluescriptII SK+, # 212205 Stratagene) with different concentrations of both Peptoids 1 and 2 in 20 μl binding buffer (5% glycerol, 10 mM Tris-HCl (pH 8.0), 1 mM EDTA, 1 mM dithiothreitol, 20 mM KCl, and 50 μg/mL bovine serum albumin). The reaction mixtures were incubated for 1 h at room temperature before running 20 μl aliquot on a 1% agarose TAE (Tris-acetate-EDTA) gel using 1× TAE buffer in the electrophoresis chamber at 125 V.

## Additional Information

**How to cite this article**: Mojsoska, B. *et al*. Peptoids successfully inhibit the growth of gram negative *E. coli* causing substantial membrane damage. *Sci. Rep.* **7**, 42332; doi: 10.1038/srep42332 (2017).

**Publisher's note:** Springer Nature remains neutral with regard to jurisdictional claims in published maps and institutional affiliations.

## Supplementary Material

Supplementary Datasets

## Figures and Tables

**Table 1:** Biological activities of Peptoids 1 and 2.

Compound/Strain	Minimal Inhibitory Concentration [MIC μg/ml (μM)]^a^				Hemolytic Concentration [μg/ml (μM)]^b^	Selectivity Index^c^	Cytotoxicity [μg/ml]^d^
	*E. coli*		*P. aeruginosa*		HC_10_	HC_10_/MIC	IC_50_
	ATCC 25922	63103 ESBL^e^	PAO1	H1027 MDR^f^			
**Peptoid 1**	16 (10.8)	16 (10.8)	16 (10.8)	2 (1.35)	128 (87)	8–68	104
**Peptoid 2**	32 (22.2)	8 (5.5)	32 (22.2)	4 (2.7)	128 (89)	4–32	110
**Indolicidin**	32 (16.8)	ND	32 (16.8)	ND	64	2–4	ND

Median MIC values representative for 3–5 replicates are given in μg/ml. ^a^Due to different molecular weights; MIC values are also given in μM, seen in brackets. ND signifies not determined.

^b^The hemolysis results are from 3 individual experiments using different blood donors.

^c^The selectivity index is the quotient of 10% hemolysis and the lowest and highest MIC of the bacterial strains.

^d^Cytotoxicity is reported as IC_50_, designated as concentration found to inhibit the metabolic activity of HeLa WT cells using the colorimetric tetrazolium salt based MTS assay.

^e^Extended spectrum beta-lactamase.

^f^multi-drug resistant *P. aeruginosa.*
