# Supplementary material for: Peptoids successfully inhibit the growth of gram negative E. coli causing substantial membrane damage
Source: Sci Rep. 2017 Feb 14;7:42332. doi: 10.1038/srep42332 (PMC5307948; doi:10.1038/srep42332)
Supplement: Supplementary Datasets [file srep42332-s1.doc]

**Peptoids successfully inhibit the growth of gram negative *E. coli* causing substantial membrane damage**

Biljana Mojsoska^a^, Gustavo Carretero ^a,b^, Sylvester Larsen^a,c^, Ramona Valentina Mateiu^d^ and Håvard Jenssen^a^

*Department of Science and Environment*, *Roskilde University, Universitetsvej 1, Postboks 260,4000 Roskilde, Denmark^a^,* Department of Biochemistry, Institute of Chemistry. University of São Paulo, São Paulo, Brazil*^b^* , Department of Clinical Immunology, Naestved Hospital, Naestved, Denmark^c^, DTU CEN, Center for Electron Nanoscopy, Technical University of Denmark, Kgs Lyngby, Denmark^d^

Corresponding Author: [*jenssen@ruc.dk*](mailto:jenssen@ruc.dk)

SUPPLEMENTARY DATA

**y = 0,108x – 0,3324**

**R^2^ = 0,985**

Figure 1. Standard curve for live/dead quantification assay


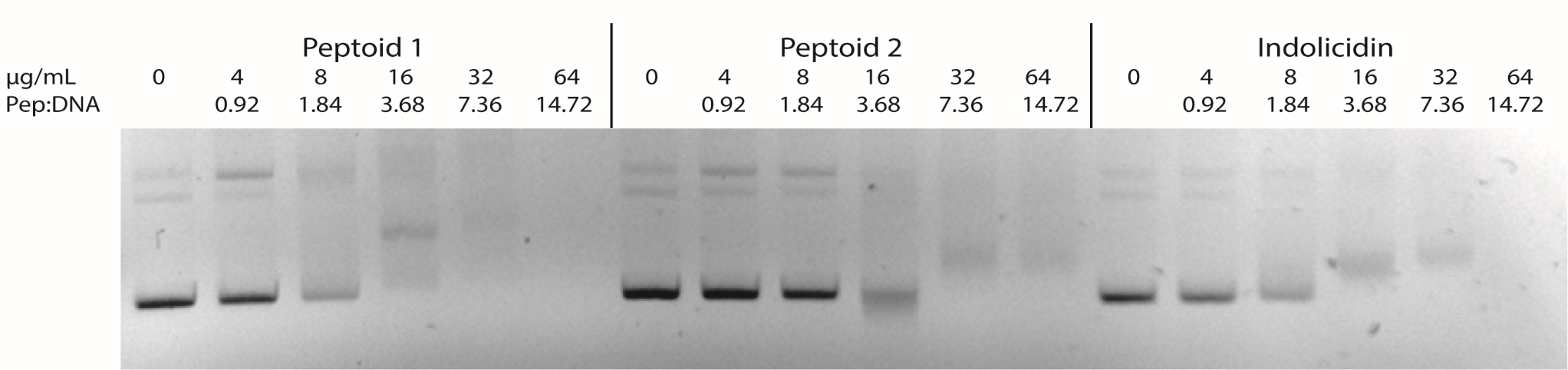


Figure 2. Peptoid interaction with bacterial plasmid DNA. Binding of Peptoids 1 and 2 was assays by measuring the degree of migration of plasmid DNA (100 ng, pBluescriptII SK+, # 212205 Stratagene) upon interaction with different concentrations of Peptoids 1 and 2. DNA and Peptoids 1 and 2 were incubated for 1h at room temperature before electrophoresis on a 1 % agarose gel.
